# P/Key: PUF based second factor authentication

**DOI:** 10.1371/journal.pone.0280181

**Published:** 2023-02-09

**Authors:** Ertan Uysal, Mete Akgün

**Affiliations:** 1 Medical Data Privacy and Privacy-Preserving ML on Healthcare Data, Department of Computer Science, University of Tübingen, Tübingen, Germany; 2 Institute for Bioinformatics and Medical Informatics, University of Tübingen, Tübingen, Germany; 3 Department of Computer Engineering, İzmir Institute of Technology, İzmir, Turkey; University College of Engineering Tindivanam, INDIA

## Abstract

One-time password (OTP) mechanisms are widely used to strengthen authentication processes. In time-based one-time password (TOTP) mechanisms, the client and server store common secrets. However, once the server is compromised, the client’s secrets are easy to obtain. To solve this issue, hash-chain-based second-factor authentication protocols have been proposed. However, these protocols suffer from latency in the generation of OTPs on the client side because of the hash-chain traversal. Secondly, they can generate only a limited number of OTPs as it depends on the length of the hash-chain. In this paper, we propose a second-factor authentication protocol that utilizes Physically Unclonable Functions (PUFs) to overcome these problems. In the proposed protocol, PUFs are used to store the secrets of the clients securely on the server. In case of server compromise, the attacker cannot obtain the seeds of clients’ secrets and can not generate valid OTPs to impersonate the clients. In the case of physical attacks, including side-channel attacks on the server side, our protocol has a mechanism that prevents attackers from learning the secrets of a client interacting with the server. Furthermore, our protocol does not incur any client-side delay in OTP generation.

## Introduction

The proliferation of Internet services means that more and more companies and individuals are doing business online. However, because the internet is accessible to everyone, the security problems faced by individuals and companies can have increasingly serious consequences. According to the statistics from Norton [1], more than half of all consumers have experienced cybercrime.

User authentication is the most important cybersecurity solution used to establish trust between users (devices) and servers. Authentication is the process of proving one’s identity while trying to access a system [2]. The clients are registered by the authentication server in the initialization step of the authentication protocols. If the client is registered in the system in the initialization part, it is granted access to the system. After successful authentication, the system checks whether a client is authorized for the desired event, e.g., by looking at the authorized entity list. Authentication is also vital for systems in which trust between devices and servers are needed such as in the Internet of Things (IoT) in which physical devices connecting to the network collect and share data to each other [3–5].

Authentication mechanisms can be classified into two groups: Single Factor Authentication (SFA) and multi-factor authentication (MFA). SFA systems are the simplest form of authentication mechanism [6]. In SFA, the client is authenticated by the system with a single secret. Authentication is generally provided with a username and password in SFA. A one-time password or facial recognition system may also serve as a single factor for authentication [7]. SFAs are preferred because of their easy deployment [8, 9]. However, passwords that are widely used as single-factor authentication provide what is considered a low level of security. This low level of security is due to the low entropy of passwords [10], possible database attacks [11], password reuse for the same or different services [12], and phishing attacks [13].

To protect users from attacks against password-based single-factor solutions, service providers offer second-factor authentication mechanisms which are a sub-branch of MFAs [14–16]. This second-factor authentication is typically based on QR codes, push notifications, or one-time passwords generated using a hardware token or a phone app. MFAs, which generally find use in systems with advanced security measures, aim to increase security by combining various forms of authentication factors in addition to the single factor when the user logs into the system [17–20]. Other factors that are independent of the first factor can be biometric features, OTP, security questions, or dedicated hardware tokens.

Second-factor authentication is provided in many ways, such as password-face recognition, and password-OTP pairs. Applications such as Google Authenticator [21] and DUO [22] generate time-based OTPs and provide a second-factor authentication mechanism integrated with different applications. OTP is often used as the second factor in authentication protocols. Being one-time use creates extra security as it provides instant use. As a first factor, static passwords are generally used. Many second factor authentication mechanisms have been proposed using OTP. Huszti and Oláh present a OTP based second factor authentication scheme using Merkle tree [23, 24]. Shivraj et al. proposed a one-time password mechanism using elliptic curve cryptography scheme and Lamport’s OTP algorithms [25]. Time-based one-time password (TOTP) is one of the widely used second-factor authentication mechanisms [26]. In TOTP, secrets are stored both on server and client side [26–29]. Therefore, once attackers access the server, they are able to capture the secrets of the registered clients. Thus, attackers can act as a client and be authenticated by the system. This risk has been encountered not only theoretically but also in practice. RSA and Linode companies got hacked, and clients’ secrets stored on their servers were stolen [30]. Since the direct storage of the keys on the server in TOTP poses a security risk, hash-chain-based mechanisms have been proposed [29, 31]. Each value in a hash-chain is used to compute different OTPs. However, the number of OTPs produced in hash-chain mechanisms is limited. After the OTPs are depleted, re-initialization of the protocol is required for OTP generation. The number of OTPs produced depends on the length of the hash-chain. If the hash-chain is kept long, the number of OTPs will increase, and the verification time will be delayed. If not, due to short hash-chains the requirement of re-initialization very often interrupts the authentication flow. The success of second-factor authentication mechanisms is evaluated by the level of security they provide, as well as the latency caused by the computational load. Designing second-factor authentication protocols that will not cause any extra computation costs in OTP generation due to providing security against server-side compromise is an open problem that needs to be addressed.

### Our contributions

In this paper, we propose a second-factor authentication mechanism called P/Key. We improve the security of standard TOTP systems against server-side compromise attacks by using Physically Unclonable Functions (PUFs) on the server side. PUF is a unique digital fingerprint of each device, originating from the production of microprocessor and semiconductor devices. PUF produces different values for each device, as it is a result of the differences that occur as a result of the production of the devices. Detailed information about PUFs is given in the Physical Unclonable Function section. The use of PUF on the server side ensures that the secret values of the clients are stored securely on the server against unauthorized access by attackers. Instead of storing clients’ secrets in plain-text form, challenges that produce clients’ secrets when applied to PUFs are stored on the server in masked form. Thus the attacker can not learn the challenges without knowing their masks in case of gaining unauthorized access to the server. During authentication, the client’s secrets are generated with the PUF and are deleted immediately after authentication. In the case of physical attacks on the server side, the PUF’s characteristics are affected, and the PUF will start behaving differently from its normal behavior. Thus attackers can not learn the clients’ secrets. In our solution, each client has two secrets and they are not present in the server’s memory at the same time during the authentication. Thus the attacker performing a cold-boot attack can learn only one of the client’s two secrets. These show that our second-factor authentication protocol is *secure against server-side compromise* as well as *physical attacks* on the server. Furthermore, we propose solutions to make corrupted PUFs available again and to determine when to direct users to re-initialization to avoid possible security vulnerabilities.

The rest of the article is as follows: In Section II, we review previously proposed second-factor authentication mechanisms and their advantages and disadvantages. In Section III, the PUF concept, working principle, and types are mentioned. In Section IV, P/Key: PUF-based second-factor authentication protocol (P/Key) is presented. In Section V, the security of the P/Key protocol is analyzed. In Section VI, our protocol is compared with previously proposed protocols and discussed according to criteria. Lastly, we bring our research to a conclusion in Section VII.

## Related work

In this section, we review IoT Authentication protocols, TOTP, S/Key, T/Key and PUF-based authentication protocols.

With the increase in IoT devices, new threats are emerging in the authentication process. It is estimated that there are more than 10 million vulnerable IoT devices [32]. Computational cost and effiency, if needed privacy preserving of user are criteria in IoT authentication [33]. Based on these problems, studies have been presented to increase authentication security in IoT devices. Fan et al. proposed a new authentication mechanism that improves IoT device security with blockchain technology [34]. Kang et al. proposed a lightweight authentication mechanism to eliminate security vulnerabilities in room services [35]. In order to provide security in cloud computing that is components of IoT, Moghaddam et al. have introduced an authentication scheme [36]. Vinoth et al. proposed an anonymous authentication and key sharing scheme for Medical IoT [37].

The advancement of IoT has also popularized the use of one-time passwords (OTP) in authentication protocols. Because lightweight communication protocols can be established between OTP servers and devices. In OTP mechanisms, the password is generated based on seed and moving factors [38]. Moving factor in HMAC one-time password (HOTP) is a counter value [39]. Counter value provides different OTPs to be produced within the HMAC function with a fixed seed value. When authentication occurs, the counter value is incremented by one on both server and client sides in the HOTP mechanism [39]. Thus, it is ensured that both parties generate the same hash values. However, in cases where the client does not send the generated password to the server, there may be a synchronization problem between the client and server counter values. Although the server checks the passwords sent by the client with more than one hash value in the window range, in case of synchronization problems, re-initialization is required between the client and server. Brute force attack poses a threat in HOTP mechanisms where the window size is wider [38].

In the time-based one-time password (TOTP) mechanism, time is a moving factor [26]. TOTP is a protocol that allows one-time use and generates a time-based password [26]. This protocol is executed over the shared secret key stored on both server and client. Passwords are valid for certain periods such as thirty seconds or one minute. Passwords are generated by performing the HMAC operation on the shared secret and time period. Since the client and server generate a password using a shared key, if unauthorized people obtain the shared key, they can generate new OTPs. TOTP is used in software-based second-factor one-time password applications such as Google Authenticator [21].

S/Key protocol overcomes the shared key storage problem on the server-side [31]. S/Key generates passwords based on a hash-chain structure, and each hashing output is a password that can only be used once [31]. Only the tail of the hash-chain is stored on the server. In the authentication phase, the user sends the hash value which is the predecessor of the tail as OTP to the server. However, S/Key is not time-based. For this reason, in case of no authentication for a long time, the same password is stored on the server. S/Key scheme is designed for a small number of login operations. The number of generated passwords is directly proportional to the length of the hash-chain. However, it is not clear how the length of the hash-chain affects the security [29]. Re-initialization is needed when the passwords created with the hash functions are depleted. Since the same hash function is used in each iteration, if the hash function is known, passwords can be generated by unauthorized people. In the case of a distributed server structure, passwords must be updated in a coordinated and secure manner on each server. In this way, all servers agree on the same response [29].

T/Key protocol proposed by Kogan et al. is a combination of TOTP and S/Key [26, 29, 31]. It aims to create an extra layer of security by adding time information to OTP as an additional feature to S/Key. However, adding time information greatly increases the computational cost of password generation and verification [40]. When authentication is required, it necessitates performing hash operations from expiration time *t*_*end*_ to the current time, one for each interval. If the validity gap is taken as 30 seconds for each OTP, it is necessary to generate 2^20^ hash operations for a period of one year. Although Kogan et al. proposed checkpoints to improve the performance of the hash-chain, in the worst-case scenario, password generation and verification times are still costly [40].

Many studies have been conducted on using PUF to authenticate IoT applications [41–44]. One of the important reasons is that the use of PUF provides a solution to the problem of storing keys in IoT devices vulnerable to tampering. Thanks to the PUF, instead of storing the key in the device, when authentication is required, the key is generated in the PUF. Yoon et al. proposed an authentication mechanism based on the usage of PUF on the client-side [41]. In [42], a PUF-based key agreement scheme has been presented for IoT devices. Wallrabenstein presented a low-cost tamper resistance authentication protocol using PUF in 2016 [43]. However, this protocol does not generate OTP and is not a second-factor mechanism. In addition to these studies, Bicakci and Baykal proposed an OTP mechanism based on asymmetric cryptography [45] with PUF. However, key generation and verification steps have higher computational costs than other protocols [46].

## Physically unclonable function

Physical Unclonable Functions (PUF) is a device-specific digital fingerprint that consists of differences in the manufacturing processes of semiconductors. These differences are uncontrollable and unpredictable. PUF mechanism can occur with the help of different physical materials such as optical materials, and RAMs on microchips. Uncontrollable temperature, electromagnetic wave, and voltage in the production processes on these physical materials cause the devices to form their own digital fingerprints and lead to each device being different at the micro level. PUFs rely on a challenge-response mechanism. When the physical structure is stimulated, the PUF produces unpredictable responses as a result of differences in this microstructure. While the stimulation of the physical structure is called the challenge, the result of the physical structure against this stimulation is called the response.

A PUF instance needs to have the following properties [47, 48]:

- Robustness: Responses of the PUF to the same challenge values at different times need to be the same or correctable with helper functions.
- Unclonability: A PUF structure can not be copied or imitated. It is not possible to perfectly emulate the physical conditions of one PUF for a different PUF instance.
- Unpredictability: Even if a sufficient number of challenge-response pairs has been obtained, it is not possible to predict beforehand the response generated in the PUF against a given challenge value.
- Tamper-evident: Any unauthorized access attempt to the PUF causes its behavior to change and accordingly generate different challenge-response values.

PUF can be implemented as means of low-cost hardware security by leveraging the unique inherent randomness of a device. Since this randomness in the microstructure may serve as a device-specific key or ID, the PUF mechanism can be applied in systems that require high security. The key of the device can be generated with answers corresponding to certain challenge values. The unique key of the same device must always be reproducible with the PUF. Against the same challenge values in the same device, PUF is expected to produce the same response, but this may not be possible due to changing physical conditions. Especially the varying noise level affecting the PUF may be a big challenge. Environmental factors such as temperature, pressure, magnetic field or power fluctuations are other factors that change the behavior of the PUF. These factors lead to the degradation of the “digital fingerprint” characterization representing the PUF. Incorrect bits occurring in the PUF need to be corrected. Otherwise, responses consisting of PUF become meaningless, and the responses cease to be device-specific. Multiple fuzzy extractor techniques may serve to correct corrupted bits [49–51]. Taniguchi et al. proposes the new soft-decision fuzzy extractor to solve instability caused by power fluctuation [51]. Aung et al. obtained stable bits using the Data Remanence algorithm on the SRAM PUF [50].

One of the major attack mechanisms that pose the threat to PUF mechanisms is “side-channel attacks”. Due to increasing security threats of side-channel attacks, defense mechanisms have become an increasingly important topic in PUF research, and some methods have been applied to PUF design to prevent side-channel information leakage [52]. For example, Yuan Cao proposed a RO PUF to protect against electromagnetic side-channel attacks [52, 53]. With the increasing emphasis on side-channel attacks by PUF designers and the combination of different defense techniques, traditional attack methods become increasingly obsolete [52]. Another significant mechanism of dealing with PUFs is “modeling attack” [54–56]. These attacks on PUFs rely on creating a dataset from challenge-response pairs (CRP) and building a new machine learning model from these CRPs. However, studies have shown that strong PUFs that are resistant to modeling attack are possible [57, 58].

PUF has been used for key generation [59, 60], key sharing [61], group key establishment [62], and IP protection [63, 64] in hardware security area. Also, PUF has become a viable work topic for IoT security and privacy due to resource constraints and access difficulties of IoT devices and found a wide variety of uses in the field [65–67]. Maurya and Bagchi propose a unilateral factor authentication mechanism for use in RFID systems [65]. Implementing PUF-based authentication schemes that ensure reasonable security of RFID tags under resource constraints is an effective way to avoid RFID deployment concerns. In a different field, due to resource constraints in IoT meters, Boyapally et al. proposed a PUF-based authentication mechanism that performs cryptographic operations on the server to provide secure communication between smart meters at consumers and the servers at the utility operators [66]. There are also studies on the use of PUF in the authentication and key-sharing processes of sensors in the wireless sensor network (WSN) [67].

PUFs can be implemented without taking up much space on hardware. It may be deployed even without using any extra equipment. To our knowledge, PUFs are not widely available to end-user on personal computers. In [68], the presence of PUFs in CPUs and GPUs was investigated. It was stated that CPU manufacturers could introduce the PUF feature on their devices by making minor changes in the hardware features. Intel employed PUF in its SGX-enabled processors [69, 70]. However, there is no commercial CPU or GPU having an integrated PUF that users can access. FPGA producers have already used PUF technology in their products. For example, Intel Stratix 10 FPGAs [71] and Xilinx UltraScale+ FPGAs [72] are equipped with PUF. These FPGA products can be easily integrated into servers and used as a PUF source. This allows our solution to be run on powerful servers.

## PUF-based second factor authentication

We propose an authentication protocol that utilizes PUFs to create a second factor. The protocol offers a time-based second factor, as in the TOTP [26]. As mentioned earlier, TOTP stores clients’ secrets on the server in plain text. However, in our proposed protocol, they are not stored on the server. PUFs are used as secure storage for them. The secrets are generated during the authentication request on the server side and deleted after use. Thus, if the server is compromised, the attacker cannot obtain them. Furthermore, our protocol is resistant to physical attacks, including side-channel attacks, according to the referred attack analysis in the previous section for the specification of general and strong PUFs. This means the physical attacker can not learn two secrets belonging to a client at the same time, as they are revealed to the server’s memory only during the protocol execution.

### Assumptions

We have the following assumptions:

- Sadeghi [73] defined the idealized behavior of PUF: it produces the same response against the same challenge value at different times and, in case of any tampering attempt on the server, PUF is destroyed and shows different characteristics than before. We assume that the ideal PUF meeting this definition is used in the protocol.
- The client and server have a synchronized clock to calculate the time period [74]. They agree beforehand on the time interval, *I*, required to calculate the number of elapsed periods.

### Protocol description

The proposed second-factor authentication protocol has two phases: initialization and authentication. The notations used in the protocol description are given in Table 1. We assume that the server has *n* different PUFs.

**Table 1:** P/Key notation table.

Symbol	Description
*s*_1_	*s*_1_ represents the challenge seed value that the client sends to the server to generate the first key.
*s*_2_	*s*_2_ represents the challenge seed value that the client sends to the server to generate the second key.
*k*_1_	*k*_1_, (i.e., *P*(*s*_1_)) is the first key value of the client. It is obtained by putting the client seed value *s*_1_ in the PUF.
*k*_2_	*k*_2_, (i.e., *P*(*s*_2_)) is the second key value of the client. It is obtained by putting the client seed value *s*_2_ in the PUF.
*a*_1_	*a*_1_ is a random value that is chosen by the client
*a*_2_	*a*_2_ is a random value that is chosen by the client.
*c*_1_	*c*_1_ is a random value that is chosen by the server.
*c*_2_	*c*_2_ is the random value that is chosen by the server.
*d*_1_	*d*_1_ is the partial seed of one of the client’s secret.
*d*_2_	*d*_2_ is the partial seed of one of the client’s secret.
*r*	*r* is a random value chosen by the client.
*I*	*I* is the time interval between two timestamps. It also determines the validity period of OTP.
*M*_1_	*M*_1_ is the first OTP.
*M*_2_	*M*_2_ is the second OTP.
*P*	PUF. {0, 1}^*l*^ → {0, 1}^*l*^
⊕	XOR operator
∈	∈ is elements of operator
*H*	Hash function. {0, 1}^*l*^ × {0, 1}^*l*^ → {0, 1}^*l*^.

#### Initialization

**Fig 1:**
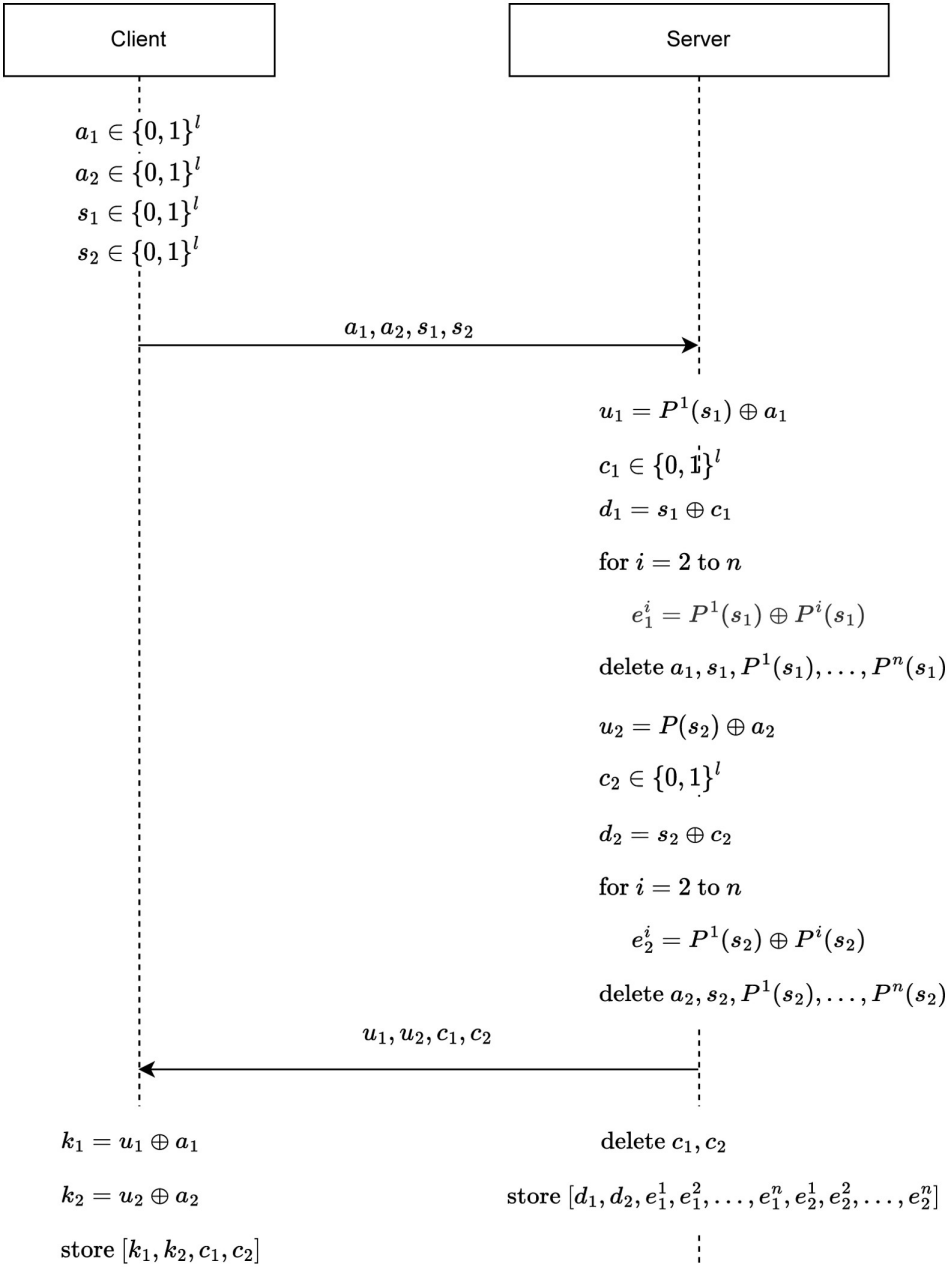
P/Key: Initialization scheme.

Initialization is a process in which a client and server agree on common secrets. They will be used to authenticate the client that wants to benefit from a service provided by the server. In the proposed initialization protocol which is depicted in Fig 1, a client chooses four different random values *a*1, *a*2, *s*1, *s*2 and sends them to the server. The server gets these random values and performs the following steps in order:

1. The server gets *s*_1_ challenge seed value which is sent by the client and put it in PUF *P*^1^. The response value produced by *P*^1^ is the secret *k*_1_ of the client. Then XOR operation is performed between *P*^1^(*s*_1_) value and *a*_1_ value. The XOR operation hides the secret *k*_1_ within *u*_1_.
2. Server chooses an *l*-bit random value *c*_1_.
3. The server performs an XOR operation between the client’s secret seed value *s*_1_, and the random value *c*_1_ of the server. Thus, the server hides the value *s*_1_ in *d*_1_. In case the server is hacked, the attacker cannot access the secret *k*_1_ of the client. Because to reach the secret *k*_1_, the attacker must know the value of *s*_1_ and put this value in the *P*^1^ as the challenge value.
4. To be able to use other PUFs in authentication, the server computes XOR of *P*^1^(*s*_1_) and each *P*^*i*^(*s*_1_) where *i* ∈ [2, *n*] and stores the result as $e_{1}^{i}$. $e_{1}^{1}$ naturally equals 0.
5. The server deletes the values *P*^*i*^(*s*_1_) where *i* ∈ [1, *n*], *a*_1_ and *s*_1_, so that in case of an attack, the attacker cannot obtain the secret *k*_1_ of the client.
6. The server generates the secret *k*_2_ of the client with *P*^1^, and then XORes *k*_2_ with *a*_2_ value. This XOR operation hides the secret *k*_2_ within *u*_2_.
7. The server chooses an *l*-bit random value *c*_2_.
8. The server hides the seed of the secret, *k*_2_, by performing an XOR operation between the seed value, *s*_2_, and the random value *c*_2_. Thus, in case the server is hacked, and unauthorized people gain database access, the attacker cannot extract the seed of the secret *k*_2_ from *d*_2_.
9. To be able to use other PUFs in authentication, the server computes XOR of *P*^1^(*s*_2_) and each *P*^*i*^(*s*_2_) where *i* ∈ [2, *n*] and stores the result as $e_{2}^{i}$. $e_{2}^{1}$ naturally equals 0.
10. The server deletes the values *P*^*i*^(*s*_1_) where *i* ∈ [1, *n*], *a*_2_ and *s*_2_ in order to prevent the attacker from accessing the secret *k*_2_.
11. The server sends *u*_1_, *u*_2_, *c*_1_ and *c*_2_ values to the client. *u*_1_ and *u*_2_ hide the secrets *k*_1_ and *k*_2_, respectively. These values are hidden by the XOR operation as mentioned above. *c*_1_ and *c*_2_ values are also sent to the client so that the client’s *s*_1_ and *s*_2_ values can be generated on the server-side in the authentication phase.
12. The server deletes *c*_1_ and *c*_2_ values after sending them to the client. If these values are not deleted and stored in the server’s database, the attacker having the credentials of the server can obtain the values *s*_1_ and *s*_2_ using the equations *s*_1_ = *d*_1_ ⊕ *c*_1_ and *s*_2_ = *d*_2_ ⊕ *c*_2_. After obtaining the *s*_1_ and *s*_2_ values, it can obtain the secrets *k*_1_ and *k*_2_ by evaluating *s*_1_ and *s*_2_, respectively, with the PUF.
13. The server stores $\left[d_{1},d_{2},e_{1}^{1},e_{1}^{2},\ldots ,e_{1}^{n},e_{2}^{1},e_{2}^{2},\ldots ,e_{2}^{n}\right]$ that are generated for each registered client along with the client’s descriptive and complementary information.

At the end of the initialization phase, the correctness test is performed for all PUFs in the system. If even one of the PUFs fails the correctness test, the initialization is performed again. Corrupted PUFs are marked for already defined clients in the system.

#### Authentication

In the proposed authentication protocol, the client computes two OTPs that are valid for a certain period. Our protocol requires a synchronized clock between the client and the server like other TOTP protocols. When authentication is needed, the client calculates the elapsed time and uses it in the computation of OTPs.

In the calculation of OTPs, a random value *r* is used. *r* is used to make two OTPs to be dependent on each other. Thus, the server that is able to generate the secrets of the client can extract the random value *r* from the first OTP *M*_1_ and use it in the verification of the second OTP *M*_2_.

Each client secret is used to calculate only one OTP. This means one is used to calculate *M*_1_ and the other is used to calculate *M*_2_. The server uses the PUF to generate the secrets of the client sequentially to verify the authentication request of the client. In the case of physical attacks (e.g. side-channel attacks), since the characteristics of the PUF change, at least one of the PUF execution will behave differently and incorrect key values will be generated. For this reason, the server will not be able to verify the client.

The client registers to the server in the initialization phase and obtains the *s*_1_, *s*_2_, *c*_1_ and *c*_2_ values required for authentication from the server.

**Fig 2:**
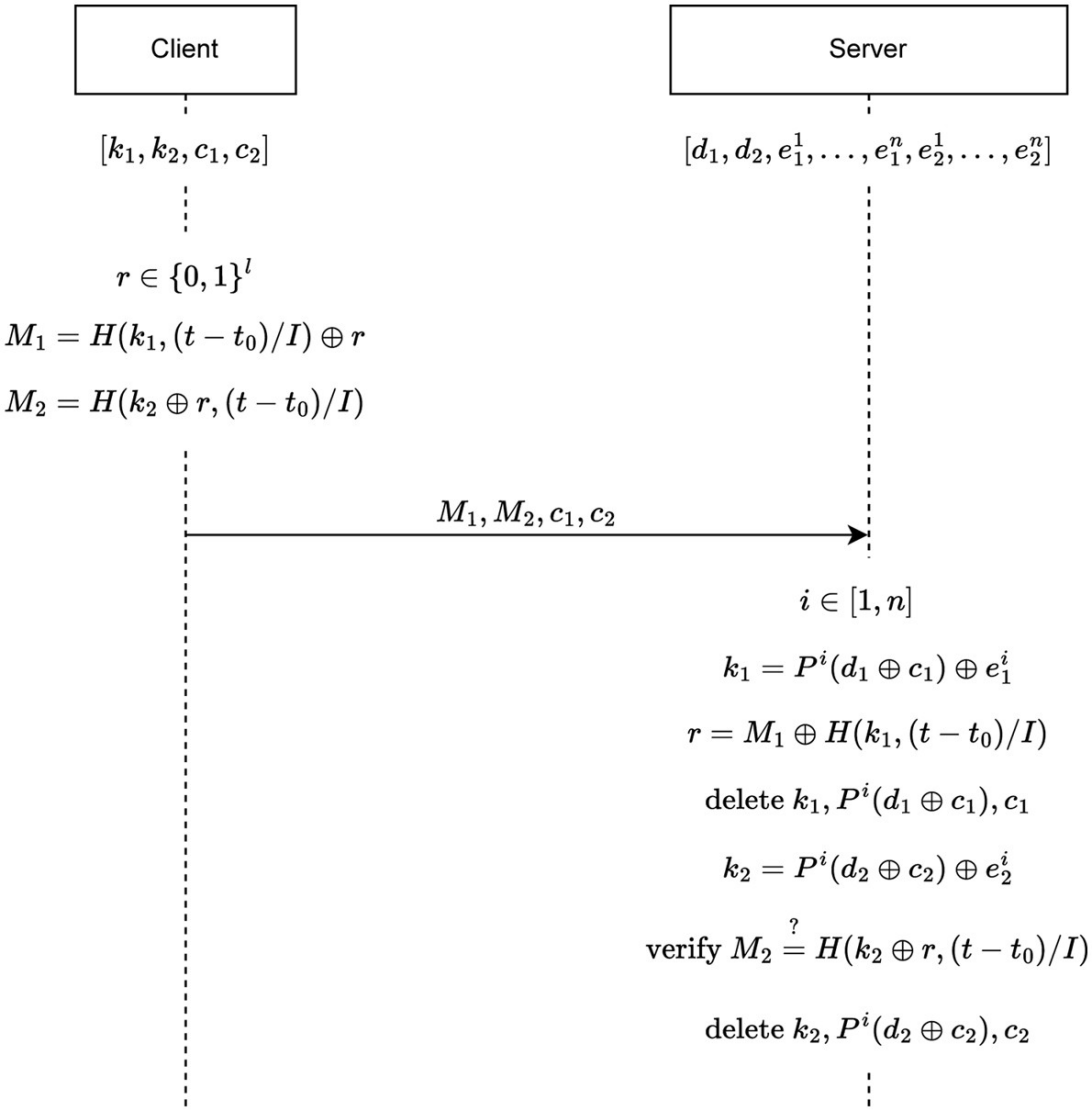
P/Key: Authentication scheme.

The authentication protocol is depicted in Fig 2 which only shows the calculations required for the validation of OTPs using PUF by the server. A detailed explanation of how the PUF recovery and redirecting to the initialization process takes place on the server during authentication is given in Algorithm 1. The steps of the authentication protocol are as follows:

1. The client generates two OTPs and sends them to the server. The steps that the client has to do are the followings:
  1. The client chooses *l*-bit random value *r*.
  2. The time period that OTPs will be valid is calculated. The current time is subtracted from the time *t*_0_, and the result is divided by interval *I* to calculate the valid time period.
  3. In this step, the secret value *k*_1_ is hashed with the time period calculated in the previous step. Then, the XOR operation is performed with the result and the *r* value. Thus, the first OTP is calculated. In this way, it is ensured that different values are produced in each authentication request, even if it is in the same time period.
  4. In the calculation of the second OTP, *M*_2_, firstly, XOR operation is performed with the *k*_2_ value and *r*. The result is hashed with the time period.
  5. *M*_1_, *M*_2_, *c*_1_ and *c*_2_ values are sent to the server as an authentication request.
2. After receiving two OTPs, the server verifies them to identify the client. The steps that the server has to do are the followings:
  1. The server randomly selects a PUF. If the selected PUF is not working correctly, the server will randomly select another PUF. The server continues this selection process until it finds a correctly working PUF. The selected PUF is represented by *P*^*i*^.
  2. The server calculates the seed *s*_1_ the client by performing an XOR operation between the *c*_1_ value that the client sends and the *d*_1_ value it stores. It gets the secret *k*_1_ by putting *s*_1_ value in *P*^*i*^.
  3. The server hashes the time period with the secret *k*_1_ obtained in the previous step. Then, an XOR operation is performed between the *M*_1_ value sent by the client and the hash result. Thus, the random value *r* is obtained. *r* value is authentication specific and different for each authentication request. The important point is that the server extracts the *r* value in the same time period as the client generated. If the *r* value is extracted after a long time has passed, the *r* value obtained will not be the same as the one produced by the client, since the time period will change.
  4. The server deletes the *k*_1_, *P*^*i*^(*d*_1_ ⊕ *c*_1_), *c*_1_ values. The reason for this is to ensure that in case of an attack in further steps, the attacker cannot learn the secret *k*_1_.
  5. The server calculates the seed *s*_2_ of the client by performing XOR operation between the *c*_2_ and *d*_2_. It gets the secret *k*_2_ by putting *s*_2_ value in *P*^*i*^.
  6. The server has the necessary *r* and *k*_2_ values to generate the *M*_2_ message. As in the client side, it generates the OTP $M_{2}^{*}$ and compares it with *M*_2_. If *M*_2_ and $M_{2}^{*}$ are equal, the server authenticates the client.
  7. The server deletes the *k*_1_, *P*^*i*^(*d*_1_ ⊕ *c*_1_), *c*_1_ values. The reason for this is to ensure that in case of an attack in further steps, the attacker cannot learn the secret *k*_2_.

**Algorithm 1**: Detailed Authentication Phase with PUF Recovery on Server Side

/* $U_{l}$ is a client wanting to access the system */

**while** *True* **do**

 *P*^*i*^ ∉ in the list of corrupted PUFs for $U_{l}$;

 **if** *P*^*i*^ *is not corrupted* **then**

$k_{1}=P^{i}(d_{1}⊕c_{1})⊕e_{1}^{i}$ ;

  **foreach** *P*^*j*^ ∈ *in the list of corrupted PUFs for* $U_{l}$ **do**

$e_{1}^{j}=P^{j}(d_{1}⊕c_{1})⊕k_{1}$ ;  // The corrupted PUF *P*^*j*^ is made to derive the *k*_1_ of $U_{l}$.

  **end**

  *r* = *M*_1_ ⊕ *H*(*k*_1_, (*t* − *t*_0_)/*I*);

  delete *k*_1_, *P*^*i*^(*d*_1_ ⊕*c*_1_), *c*_1;_

  **if** *P*^*i*^ *is corrupted* **then**

   **foreach** *U* ∈ *Clients* **do**

    Mark *P*^*i*^ as corrupted in the list of corrupted PUFs for U;

   **end**

   Redirect $U_{l}$ to initialization;

   Exit loop; // *P*^*i*^ is corrupted. Thus one secret of $U_{l}$ may have been compromised. The authentication process is terminated and $U_{l}$ is redirected to initialization to determine its new secrets.

  **end**

$k_{2}=P^{i}(d_{2}⊕c_{2})⊕e_{2}^{i}$ ;

  **foreach** *P*^*j*^ ∈ in the list of corrupted PUFs for $U_{l}$ **do**

$e_{2}^{j}=P^{j}(d_{2}⊕c_{2})⊕k_{2}$ ; // The corrupted PUF *P*^*j*^ is made to derive the *k*_2_ of $U_{l}$.

   Mark *P*^*j*^ as uncorrupted in the list of corrupted PUFs for $U_{l}$;

  **end**

  **if** *M*_2_ = *H*(*k*_2_ ⊕ *r*, (*t* − *t*_0_)/*I*) **then**

$U_{l}$ is authenticated;

  **end**

  delete *k*_2_, *P*^*i*^(*d*_2_ ⊕ *c*_2_), *c*_2_;

  **if** *P*^*i*^ *is corrupted* **then**

Redirect $U_{l}$ to initialization in the next authentication request; // *P*^*i*^ is corrupted. Thus one secret of $U_{l}$ may have been compromised. $U_{l}$ is authenticated and $U_{l}$ will be redirected to initialization to determine its new secrets in the next authentication request.

   **foreach** *U* ∈ *Clients* **do**

    Mark *P*^*i*^ as corrupted in the list of corrupted PUFs for U;

   **end**

  **end**

  Exit loop;

 **else**

  **foreach** *U* ∈ *Clients* **do**

   Mark *P*^*i*^ as corrupted in the list of corrupted PUFs for U;

  **end**

 **end**

**end**

#### PUF Recovery and re-initialization of secrets

In the authentication phase, before verifying the OTPs of a client, the server checks the correctness of the PUF that it randomly chose. For this purpose, a predetermined challenge-response pair (*x*, *y*) is stored on the server for each PUF. If a PUF cannot produce the response *y* corresponding to challenge *x*, this indicates that the PUF is corrupted. To make the corrupted PUF *P*^*j*^ become available again, new $e_{1}^{j}$ and $e_{2}^{j}$ values are calculated for a client whose authentication request is received. First, the server finds a PUF that is uncorrupted and computes *k*_1_ of the client. The server learns a new $e_{1}^{j}$ for the corrupted PUF *P*^*j*^ by computing *P*^*j*^(*d*_1_ ⊕ *c*_1_) ⊕ *k*_1_. Second, the server computes *k*_2_ of the client using the uncorrupted PUF. The server learns a new $e_{2}^{j}$ for the corrupted PUF *P*^*j*^ by computing *P*^*j*^(*d*_2_ ⊕ *c*_2_) ⊕ *k*_2_. Thus, the re-initialization of the corrupted tag for the client is done by performing two additional computations in the authentication phase.

During authentication, only one of client’s secrets can be obtained by the attacker with a cold boot attack. In another authentication session where another PUF is used, the attacker can obtain the other key of the same client with a cold boot attack. Thus the attacker can impersonate the client. For this reason, clients whose one key is likely to be learned by the attacker are detected during authentication and directed to initialization in the next authentication request. These clients are detected by PUF correctness tests to be performed before the generation of *k*_2_ and after the deletion of *k*_2_. The former correctness test detects that the attacker obtained *k*_1_ and the latter one detects that the attacker obtained *k*_2_. How the PUF recovery process takes place on the server side during the authentication phase is explained in detail in Algorithm 1.

## Security analysis

In this section, we will present the security analysis of our authentication protocol.

### Threat model

We assume that the user device (client) meets the required security requirements, and does not carry any malware so that the user session cannot be hijacked by an attacker. We also assume that the communication channel between the user device and the server is protected by TLS [75], thus man-in-the-middle (MITM) attacks are not possible. All TOTP schemes are vulnerable to online phishing attacks, where users’ short-term one-time passwords are compromised. However, the time limit of one-time passwords makes it difficult to carry out an attack using them.

We assume that adversaries are able to access the server multiple times and obtain the necessary information to authenticate the clients. For our protocol, the information received from the compromised server is the seeds of the clients’ secrets and the passwords that can be retrieved from the memory during the execution of the protocol for a specific client.

We assume that attackers with physical access to the server can perform side-channel attacks [76], especially cold boot attacks, to extract client passwords and secrets.

### Formal definition of one-time password protocol

A one-time password protocol is defined by the following procedures:

-
$PPGen(1^{s})$ : is an algorithm that outputs the password length *l* with the given security parameter *s*.
-
KeyGen(l) : is a probabilistic polynomial-time algorithm that outputs the secrets *k*_1_ and *k*_2_ of the prover and the internal state of the verifier consisting of partial seeds *d*_1_ and *d*_2_ of the prover’s secrets and masks $e_{1}^{1},e_{1}^{1},\ldots ,e_{1}^{n}$ and $e_{2}^{1},e_{2}^{1},\ldots ,e_{2}^{n}$ of PUFs in the system.
-
$Prover(t,k_{1},k_{2})$ : is a polynomial time algorithm that takes the time *t* and the prover’s secrets *k*_1_ and *k*_2_ and outputs the one-time passwords *M*_1_ and *M*_2_ and masks *c*_1_ and *c*_2_.
-
$Verifier(t,d_{1},d_{2},M_{1},M_{2},c_{1},c_{2},e_{1}^{1},e_{1}^{1},\ldots ,e_{1}^{n},e_{2}^{1},e_{2}^{1},\ldots ,e_{2}^{n})$ : is a polynomial time algorithm that takes as input the time *t* and the verifier’s internal state *d*_1_, *d*_2_, $e_{1}^{1},e_{1}^{1},\ldots ,e_{1}^{n}$ and $e_{2}^{1},e_{2}^{1},\ldots ,e_{2}^{n}$, and one-time passwords *M*_1_ and *M*_2_. It outputs accept or reject based on whether one-time passwords are verified successfully.

In order to prove the correctness, our protocol must output “accept” for every $Verifier(t,d_{1},d_{2},M_{1},M_{2},c_{1},c_{2},e_{1}^{1},e_{1}^{1},\ldots ,e_{1}^{n},e_{2}^{1},e_{2}^{1},\ldots ,e_{2}^{n})$ call, where *t* is monotonically increasing and, *M*_1_ and *M*_2_ are produced with $Prover(t,k_{1},k_{2})$.

PPGen and KeyGen procedures correspond to the initialization phase of our protocol. Prover and Verifier procedures correspond to the authentication phase of our protocol.

### Adversary model

The adversary is mainly defined by specifying the actions she is allowed to take (the oracles she can query), the purpose of her attack (the definition of the game), and the way she interacts with the server and clients.

An adversary is an algorithm that can run the following oracles

-
Launch : enables the client to start a new protocol instance *π* at time *t*.
-
SendServer(m,π,t) : sends a message *m* to the server in a protocol instance *π* for the time *t*. Then, it receives the message *m*′ as an answer.
-
Result(π) : returns 1 if the server verifies a client, and 0 otherwise at the end of the protocol session *π*.
-
CorruptServer() : corrupts the server and gets its internal state.

### Analysis

In this section, we analyze the security of the proposed protocol.

**Definition 1 (Hash Function)**. *Let l* ∈ **N** *be a security parameter*, *γ*, *κ* ∈ **N** *be polynomially bounded in l*. *A hash function H is defined as* {0, 1}^*γ*^ → {0, 1}^*κ*^ *with the following basic requirements*:

1. *For a given output y*_*i*_, *it is computationally infeasible to find an input x*_*i*_ *satisfying h*(*x*_*i*_) = *y*_*i*_.
2. *It is computationally infeasible to find a pair* (*x*_*i*_, *x*_*j*_) *satisfying x*_*i*_ ≠ *x*_*j*_ *and h*(*x*_*i*_) = *h*(*x*_*j*_).
3. *Any probabilistic polynomial time adversary who queried H for a polynomial number of times can distinguish the output of H with at most negligible probability*.

**Definition 2 (Physically Unclonable Function (PUF) [73])**. *Let l* ∈ **N** *be a security parameter*, *γ*, *κ* ∈ **N** *be polynomially bounded in l*. *An ideal PUF P is defined as* {0, 1}^*γ*^ → {0, 1}^*κ*^ *that has the following parameters*:

1. *For all c* ∈ {0, 1}^*γ*^ *and all pairs* (*r*_*i*_, *r*_*j*_) ∈ [*P*(*c*)]^2^, *it holds that probability Pr*[*r*_*i*_ = *r*_*j*_] = 1.
2. *Any physical attempt to tamper with the device on which P is implemented results in the destruction of P*. *Thus P cannot be evaluated correctly anymore because its behavior has changed*.
3. *Any probabilistic polynomial time adversary who queried P for a polynomial number of times can compute the output of P with at most negligible probability*.

**Lemma 1**. *Let* A *be an adversary. The advantage of* A *of obtaining the secrets k*_1_ *and k*_2_ *by corrupting a server during the execution of the initialization protocol is negligible*.

*Proof*. We assume that there is an adversary A that can learn the secrets *k*_1_ and *k*_2_ of a client by corrupting the server during the execution of the initialization protocol. If A corrupts the server while it is not interacting with any client, A does not learn anything because the volatile memory is empty. In the case where A corrupts the server while interacting with a client, corruption time is important to determine what the attacker can learn because deletions of some values are performed two times during the protocol execution. Assume that A corrupts the server before the first deletion and obtains *a*_1_, *a*_2_, *s*_1_, *s*_2_, *u*_1_, *c*_1_, *d*_1_, and $\left(e_{1}^{1},\ldots ,e_{1}^{n}\right)$. A extracts the secret *k*_1_ by computing *k*_1_ = *u*_1_ ⊕ *a*_1_ and wants to infer the secret *k*_2_. In order to infer *k*_2_, A has to simulate the PUF *P*^*i*^(.), *i* ∈ [1, *n*] but this contradicts the security of PUF (Definition 2). Assume that A corrupts the server before the second deletion and obtains *a*_2_, *s*_2_, *u*_1_, *u*_2_, *c*_1_, *c*_2_, *d*_1_, *d*_2_, $\left(e_{1}^{1},\ldots ,e_{1}^{n}\right)$, and $\left(e_{2}^{1},\ldots ,e_{2}^{n}\right)$. A computes the secret *k*_2_ = *u*_2_⊕*a*_2_ and wants to infer the secret *k*_1_. In order to infer *k*_1_, A has to expose it from *u*_1_ but *u*_1_ and *a*_1_ are random it is not possible extract *k*_1_ from *u*_1_ without knowing *a*_1_. Alternatively, A can calculate $P^{i}(d_{1}⊕c_{1})⊕e_{1}^{i}$ to get the secret *k*_1_, but this means A can simulate *P*^*i*^(.). This contradicts the security of PUF (Definition 2). As a result, A can learn *k*_1_ and *k*_2_ by corrupting the server during the execution of the initialization protocol with negligible probability.

**Lemma 2**. *Let* A *be an adversary*. *The advantage of* A *of obtaining the secrets k*_1_ *and k*_2_ *by corrupting a server during the execution of the authentication protocol is negligible*.

*Proof*. We assume that there is an adversary A that can learn the secrets *k*_1_ and *k*_2_ of a tag by corrupting the server during the execution of the authentication protocol. If A corrupts the server while it is not interacting with a client, A does not learn anything because the volatile memory is empty. In the case where A corrupts the server while interacting with a client, corruption time is important to determine what the attacker can learn because deletions of some values are performed two times during the protocol execution. Assume that A corrupts the server before the first deletion and obtains *M*_1_, *M*_2_, *c*_1_, *c*_2_, *k*_1_, *r*, *d*_1_, *d*_2_, $\left(e_{1}^{1},\ldots ,e_{1}^{n}\right)$, and $\left(e_{2}^{1},\ldots ,e_{2}^{n}\right)$. A knows the secret *k*_1_ and wants to infer the secret *k*_2_. In order to infer *k*_2_, A has to expose it from *M*_2_ but this contradicts the security of hash functions (Definition 1). A knows that $k_{2}=P^{i}(d_{2}⊕c_{2})⊕e_{2}^{i}$, *i* ∈ [1, *n*] so the other way to infer *k*_2_ is to simulate *P*^*i*^(.). This contradicts the security of PUF (Definition 2). Assume that A corrupts the server before the second deletion and obtains *M*_1_, *M*_2_, *c*_1_, *c*_2_, *k*_2_, *r*, *d*_1_, *d*_2_, $\left(e_{1}^{1},\ldots ,e_{1}^{n}\right)$, and $\left(e_{2}^{1},\ldots ,e_{2}^{n}\right)$. A knows the secret *k*_2_ and wants to infer the secret *k*_1_. In order to infer *k*_1_, A has to expose it from *M*_1_ but this contradicts the security of hash functions (Definition 1). A knows that $k_{1}=P^{i}(d_{1}⊕c_{1})⊕e_{1}^{i}$ so the other way to infer *k*_1_ is to simulate *P*^*i*^(.). This contradicts the security of PUF (Definition 2). As a result, A can learn *k*_1_ and *k*_2_ by corrupting the server during the execution of the authentication protocol with negligible probability.

**Lemma 3**. *Let* A *be an adversary. The advantage of* A *of obtaining the secrets k*_1_ *and k*_2_ *by corrupting a server during the execution of different sessions of the authentication protocol is negligible*.

*Proof*. Lemma 2 states that A who corrupts the server during an authentication session of a client can only obtain one of the secrets of that client. However, if A corrupts the server during another authentication session of the same client, she can obtain the other secret of that client. The main reason for this attack is that multiple PUFs are used in our system and all PUFs derive the same secrets for a client.

By performing PUF accuracy tests, we can detect whether the PUF used during the client’s authentication session is corrupted. The corruption of the used PUF means that at most one secret of the client may be obtained by A. To prevent A from obtaining the other secret of the client, in such cases we redirect the client to the initialization phase to change its secrets. As a result, A can learn *k*_1_ and *k*_2_ by corrupting a server during the execution of different sessions of the authentication protocol with negligible probability.

**Theorem 1**. *The proposed authentication protocol provides second-factor authentication*.

**Proof**. We assume that there is an adversary A that can generate valid *M*_1_, *M*_2_, *c*_1_ and *c*_2_ for a given client. A wins the security experiment if *M*_1_ and *M*_2_ pass the verification in the server. The communication between the client and server is secured so A cannot listen to the channel between them.

Assume that during the execution of the initialization and authentication protocols between the client and server, A calls CorruptServer() oracle to obtain the secrets of the client. This will contradict Lemma 1 stating that A can not learn the secrets of a given client by corrupting the server during the execution of the initialization protocol and with Lemma 2 stating that A can not learn the secrets of a given client by corrupting the server during the execution of the authentication protocol.

Assume that during the execution of the authentication protocol between the client and server, A call CorruptServer() oracle on different authentication sessions of the client to obtain its secrets. This will contradict Lemma 3 stating that A can not learn the secrets of a given client by corrupting the server on different authentication sessions of the client.

A performing the server corruption can obtain one-time passwords *M*_1_ and *M*_2_ created by the client. The time limitation of one-time passwords makes it difficult to perform an impersonation attack using them. This is the common problem of all TOTP protocols.

Additionally, an adversary who obtains server login credentials learns partial seeds of client secrets from the server database and can execute the PUF without breaking it. It is impossible for an attacker to learn secrets from partial seeds. The adversary who has accessed the server can learn the secrets of the clients that sent authentication requests to the server during the time when the adversary has control of the server. Since we can assume that it is very difficult to learn the login credentials of the server and that the detection time of an adversary who has accessed the server is very short, the probability of such an attack is negligible.

#### Impersonation attacks

In order to impersonate a client, an adversary A must either compute or learn valid one-time passwords *M*_1_ and *M*_2_. To compute valid *M*_1_ and *M*_2_, A needs to know the secrets *k*_1_ and *k*_2_ of the client. Lemma 1, Lemma 2, and Lemma 3 show that it is impossible to learn the secrets of any client by corrupting the server.

By hijacking the client device, A can learn *k*_1_ and *k*_2_. As we stated in our threat model, we assume that the necessary security measures are taken for the client’s device. The best countermeasure for masquerading attacks is not to store customer secrets on the client device or keep them encrypted [77].

Since the communication between the client and server is protected by TLS, it is impossible for A to monitor and eavesdrop on outgoing messages. Therefore, it is not possible to apply a replay attack and a man-in-the-middle attack to impersonate a client. A compromising the server may attempt to use previous one-time passwords to impersonate the client. A cannot utilize *M*_1_ and *M*_2_ used in previous sessions because *M*_1_ and *M*_2_ are valid in a certain time interval. Besides, *M*_1_ and *M*_2_ can only be used once hence, if they are used for authentication again, they will not be accepted by the server [78].

#### Re-initialization of second factor

In case, the client device is lost, the client who successfully passes the first-factor authentication can access the service with another authentication mechanism provided by the service provider. The client can re-enable our second-factor authentication protocol for the relevant service using our initialization protocol. Password recovery corresponds to the initialization phase of our protocol. In case the client loses his/her device or all PUFs on the server are corrupted, the client is directed to the re-initialization phase. This redirection is done via the e-mail address linked to the user account. This shows that if the adversary can pass the first-factor authentication and gain access to the legitimate user’s email account, she can initiate the initialization protocol instead of the legitimate user.

## Performance evaluation

In this section, we present the performance of the proposed scheme in terms of storage cost, communication cost, and computational cost, similar to the performance analysis done in [79]. Table 2 summarizes the costs of our protocol and other related schemes.

**Table 2:** Storage, computation, and communication costs of various protocols.

	Storage		Computation		Communication
	Client	Server	Client	Server	
TOTP	*ℓ*	*mℓ*	*H*	*H*	*ℓ*
S/Key	(log *k*)*ℓ*	*mℓ*	$\frac{\text{log}\;k}{2}H$	*H*	*ℓ*
T/Key	(log *k*)*ℓ* *	*mℓ*	$\frac{\text{log}\;k}{2}H$ *	*H*	*ℓ*
P/Key	4*ℓ*	*m*(2*n* + 2)*ℓ*	2*H*	2*H* + 2*P*	4*ℓ*

*ℓ*: the bit length of secret parameters and OTPs

*m*: the number of clients

*n*: the number of PUF instances

*k*: the size of hash chain

*H*: Hash computation

*P*: PUF computation

* Most likely larger because of arbitrary gaps in the hash chain evaluation

In our protocol, a client stores 4 values size of *ℓ*-bit and the server stores *m*(2*n* + 2) *ℓ*-bit where *n* is the number of PUF instances and *m* is the number of clients. The storage cost of our protocol on the client side is higher than TOTP. Considering the level of security provided by our protocol compared to TOTP, this difference is negligible. Our protocol has more storage costs on the server side than other protocols. This cost difference is also negligible, assuming the servers are powerful devices. For example, if *ℓ* is 160, *n* is 100 and *m* is 100, 000, 000, 404 GB of storage space is needed on the server. It is a very small storage space requirement for a system serving 100, 000, 000 clients.

Our protocol requires 2 hash computations on the client and 2 hash and 2 PUF computations on the server. The computation cost of our protocol on the client side is nearly the same or less than other protocols. The computation cost of other protocols on the server side is the computation of a single hash. This difference is negligible for powerful servers when considering the security, and the lower computation cost of it on the client side provided by our protocol.

The communication cost of our protocol is 4*ℓ*-bits. If *l* is assumed to be 160, the transmission of 80 bytes can be done easily with today’s technology. When evaluated with the communication cost of other protocols, it shows that our protocol does not have a heavy communication cost.

## Discussion

A comparison of the proposed protocol with the other three protocols is given in Table 3. Under this section, we will evaluate every feature provided in this table.

**Table 3:** Comparison of the proposed protocol with various protocols.

	TOTP	S/Key	T/Key	P/Key
Resistance to Server-Side Physical Attacks	No	Yes	Yes	Yes
Resistance to Server-Side Compromise Attacks	No	Yes	Yes	Partial*
Hash-chain Usage	No	Yes	Yes	No
Resistance to Replay Attacks (Time-based Passwords)	Yes	No	Yes	Yes

*Only the secrets of the clients that send the authentication request to the compromised server are revealed.

In the case of physical attacks, including side-channel attacks on the server for our proposed protocol P/Key, the physical structure of the PUF will change. Therefore, the server will not be able to generate the correct secrets using the PUF. Hence, the protocol is resistant to physical attacks on the server side. In the case of physical attacks in the T/Key protocol, the OTPs obtained by the attacker are insufficient for impersonation, because OTPs are time-dependent and the attacker cannot obtain the previous hash values to generate the future OTPs. In the S/Key protocol, the OTP of a client obtained by the attacker can be used to impersonate that client with high probability because the OTP is not time-dependent. But, as in T/Key, the attacker cannot obtain the previous values in the hash-chain and cannot generate future OTPs. In the TOTP mechanism, the secret of a client sending the authentication request to the server can be obtained with physical attacks and then used to impersonate the client, thus, it is accepted to be not resistant to physical attacks.

An attacker compromising the server can not obtain the secrets of the clients in our protocol as explained in detail in section IV since the seeds of the clients’ secrets are stored on the server in a hidden format. However, the attacker compromising the server can learn the secrets of the clients who at the moment send an authentication request to the server in our protocol. But, in this case, the secrets of other clients previously registered on the server are still safe. For this reason, our protocol is partially resistant to server-side compromising. In T/Key and S/Key protocols, secrets are kept hashed on the server. Thus, these protocols are also resistant to server-side compromising. But the situation is different in TOTP. The attacker who takes over the server by getting the server credentials can generate the OTP because it knows the secret of the clients and therefore which is not resistant to server-side compromise attacks.

Our P/Key protocol differs from other competitors and does not use the hash-chain structure. Because many hash operations take place on the client side, the generation of OTPs can be costly in hash-chain-based systems. In our protocol, the authentication and key generation processes are done with the PUF for each authentication and can be completed quickly with a limited number of steps. Thanks to PUF, the P/Key protocol responds quickly to authentication requests on the server side as intended.

At last, we evaluate the protocols related to replay attack possibilities for the server-side secret values. In time-based OTP protocols, secrets are periodically renewed on the server side. For this reason, the same secret is not stored on the server for a long time. TOTP, T/Key, and P/Key are time-based OTP mechanisms. However, in the S/Key protocol, the stored secret required for verification on the server is not updated until a new authentication occurs. It is only updated when it is used in the S/Key mechanism. Because of that, OTP can be valid for a long time. Storing the same secret on the server for a long time poses a security threat.

As a result, our second-factor authentication protocol keeps clients’ secrets secure even if the server is compromised because their secrets are stored on the server in masked form. It provides resistance to physical attacks because the clients’ secrets are generated by executing PUFs on the server side. The attacker can not obtain two secrets of a client at the same time. Our protocol also generates OTPs in constant time (two hash calls) on the client side because it does not depend on the hash-chain mechanism. This also speeds up the authentication process.

## Conclusion

In this paper, we presented a second-factor authentication protocol called P/Key. Our protocol is resistant to server-side compromise attacks that reveal the clients’ secrets to the attacker. Prior works such as S/Key and T/Key based on the hash-chain mechanism remain secure in case of server compromise. However, the number of OTPs to be generated is limited as it depends on the length of the hash-chain. As the length of the hash-chain increases, the number of OTPs increases, but the generation of OTPs and their verification on the server side can take longer, which slows down the authentication process. Since the number of OTPs is limited, they require re-initialization after a certain period. Our work combines the basic idea behind TOTP with the utilization of PUFs on the server side to resist server-side compromise attacks. We show that in case of compromised server and physical attacks, including side-channel attacks, the secrets of the clients remain secure. Furthermore, we showed that our protocol works with multiple PUF instances, each possibly dedicated to a different server. Usage of the multiple PUF instances ensures that our protocol continues to serve by detecting and using uncorrupted PUFs even if there are PUFs that are corrupted due to reasons such as side-channel attacks, thus exhibiting different behavior.

In future work, we would like to work on a solution to the instance when an attacker having access to the compromised server is able to learn the secrets of a client that sends the authentication request to the compromised server at the time of the attack. We also would like to work on customizing our protocol to IoT usage domains.

## References

[1] 2021 NORTON CYBER SAFETY INSIGHTS REPORT GLOBAL RESULTS;. Available at https://now.symassets.com/content/dam/norton/campaign/NortonReport/2021/2021_NortonLifeLock_Cyber_Safety_Insights_Report_Global_Results.pdf.

[2] Lamport L. Password authentication with insecure communication. Communications of the ACM. 1981;24(11):770–772. doi: 10.1145/358790.358797

[3] Laxmi AR, Mishra A. RFID based Logistic Management System using Internet of Things (IoT). In: 2018 Second International Conference on Electronics, Communication and Aerospace Technology (ICECA); 2018. p. 556–559.

[4] Wu F, Wu T, Yuce MR. Design and Implementation of a Wearable Sensor Network System for IoT-Connected Safety and Health Applications. In: 2019 IEEE 5th World Forum on Internet of Things (WF-IoT); 2019. p. 87–90.

[5] Niranjane V, Shelke U, Shirke S, Dafe S. IoT based Digital Production Counting System. In: 2022 International Conference on Electronics and Renewable Systems (ICEARS); 2022. p. 452–455.

[6] Feltner S. Single-factor authentication (SFA) vs. multi-factor authentication (MFA); 2016. Available from: https://www.centrify.com/blog/sfa-mfa-difference/#:~:text=Single%2Dfactor%20authentication%20is%20the,this%20type%20of%20authentication%20method.

[7] Benarous L, Kadri B, Bouridane A. A survey on cyber security evolution and threats: biometric authentication solutions. In: Biometric security and privacy. Springer; 2017. p. 371–411.

[8] Konoth RK, Veen Vvd, Bos H. How anywhere computing just killed your phone-based two-factor authentication. In: International conference on financial cryptography and data security. Springer; 2016. p. 405–421.

[9] Kim JJ, Hong SP. A method of risk assessment for multi-factor authentication. Journal of Information Processing Systems. 2011;7(1):187–198. doi: 10.3745/JIPS.2011.7.1.187

[10] Komanduri S, Shay R, Kelley PG, Mazurek ML, Bauer L, Christin N, et al. Of passwords and people: measuring the effect of password-composition policies. In: Proceedings of the sigchi conference on human factors in computing systems; 2011. p. 2595–2604.

[11] Cameron D. Over 560 million passwords discovered in anonymous online database; 2017.

[12] Florêncio D, Herley C, Van Oorschot PC. Password Portfolios and the {Finite-Effort} User: Sustainably Managing Large Numbers of Accounts. In: 23rd USENIX Security Symposium (USENIX Security 14); 2014. p. 575–590.

[13] Egelman S, Cranor LF, Hong J. You’ve been warned: an empirical study of the effectiveness of web browser phishing warnings. In: Proceedings of the SIGCHI Conference on Human Factors in Computing Systems; 2008. p. 1065–1074.

[14] Schneier B. Two-factor authentication: too little, too late. Communications of the ACM. 2005;48(4):136. doi: 10.1145/1053291.1053327

[15] Petsas T, Tsirantonakis G, Athanasopoulos E, Ioannidis S. Two-factor authentication: is the world ready? Quantifying 2FA adoption. In: Proceedings of the eighth european workshop on system security; 2015. p. 1–7. doi: 10.1145/2751323.2751327

[16] Wang D, He D, Wang P, Chu CH. Anonymous two-factor authentication in distributed systems: Certain goals are beyond attainment. IEEE Transactions on Dependable and Secure Computing. 2014;12(4):428–442. doi: 10.1109/TDSC.2014.2355850

[17] Scheidt EM, Domangue E. Multiple factor-based user identification and authentication; 2005.

[18] Bhargav-Spantzel A, Squicciarini AC, Modi S, Young M, Bertino E, Elliott SJ. Privacy preserving multi-factor authentication with biometrics. Journal of Computer Security. 2007;15(5):529–560. doi: 10.3233/JCS-2007-15503

[19] Banyal RK, Jain P, Jain VK. Multi-factor authentication framework for cloud computing. In: 2013 Fifth International Conference on Computational Intelligence, Modelling and Simulation. IEEE; 2013. p. 105–110.

[20] Frank M, Biedert R, Ma E, Martinovic I, Song D. Touchalytics: On the applicability of touchscreen input as a behavioral biometric for continuous authentication. IEEE transactions on information forensics and security. 2012;8(1):136–148. doi: 10.1109/TIFS.2012.2225048

[21] Authentication—Google Safety Center;. https://safety.google/authentication.

[22] Guide to Two-Factor Authentication · Duo Security;. https://guide.duo.com.

[23] Huszti A, Oláh N. A simple authentication scheme for clouds. In: 2016 IEEE Conference on Communications and Network Security (CNS). IEEE; 2016. p. 565–569.

[24] Sheik SA, Muniyandi AP. Secure authentication schemes in cloud computing with glimpse of artificial neural networks: A review. Cyber Security and Applications. 2023;1:100002. doi: 10.1016/j.csa.2022.100002

[25] Shivraj VL, Rajan MA, Singh M, Balamuralidhar P. One time password authentication scheme based on elliptic curves for Internet of Things (IoT). In: 2015 5th National Symposium on Information Technology: Towards New Smart World (NSITNSW); 2015. p. 1–6.

[26] M’Raihi D, Machani S, Pei M, Rydell J. Totp: Time-based one-time password algorithm. Internet Request for Comments. 2011; p. 685E.

[27] Uymatiao MLT, Yu WES. Time-based OTP authentication via secure tunnel (TOAST): A mobile TOTP scheme using TLS seed exchange and encrypted offline keystore. In: 2014 4th IEEE International Conference on Information Science and Technology. IEEE; 2014. p. 225–229.

[28] Park WS, Hwang DY, Kim KH. A TOTP-based two factor authentication scheme for hyperledger fabric blockchain. In: 2018 Tenth International Conference on Ubiquitous and Future Networks (ICUFN). IEEE; 2018. p. 817–819.

[29] Kogan D, Manohar N, Boneh D. T/key: second-factor authentication from secure hash chains. Proceedings of the 2017 ACM SIGSAC Conference on Computer and Communications Security. 2017. doi: 10.1145/3133956.3133989

[30] Zetter K. RSA Agrees to Replace Security Tokens After Admitting Compromise. Threat Level, Privacy, Crime and Security Online. 2011;.

[31] Haller N. RFC1760: The S/KEY One-Time Password System; 1995.

[32] Haseeb J, Mansoori M, Welch I. A Measurement Study of IoT-Based Attacks Using IoT Kill Chain. In: 2020 IEEE 19th International Conference on Trust, Security and Privacy in Computing and Communications (TrustCom); 2020. p. 557–567.

[33] Jiang Y, Zhang K, Qian Y, Zhou L. Anonymous and Efficient Authentication Scheme for Privacy-Preserving Distributed Learning. IEEE Transactions on Information Forensics and Security. 2022;17:2227–2240. doi: 10.1109/TIFS.2022.3181848

[34] Fan Q, Chen J, Deborah LJ, Luo M. A secure and efficient authentication and data sharing scheme for Internet of Things based on blockchain. Journal of Systems Architecture. 2021;117:102112. doi: 10.1016/j.sysarc.2021.102112

[35] Kang D, Lee H, Lee Y, Won D. Lightweight user authentication scheme for roaming service in GLOMONET with privacy preserving. Plos one. 2021;16(2):e0247441. doi: 10.1371/journal.pone.0247441 33635893 PMC7909710

[36] Moghaddam FF, Varnosfaderani SD, Ghavam I, Mobedi S. A client-based user authentication and encryption algorithm for secure accessing to cloud servers based on modified Diffie-Hellman and RSA small-e. In: 2013 IEEE Student Conference on Research and Developement. IEEE; 2013. p. 175–180.

[37] Vinoth R, Deborah LJ, Vijayakumar P, Gupta BB. An Anonymous Pre-Authentication and Post-Authentication Scheme Assisted by Cloud for Medical IoT Environments. IEEE Transactions on Network Science and Engineering. 2022;. doi: 10.1109/TNSE.2022.3176407

[38] n a na. OTP, TOTP, HOTP: What’s the difference?: OneLogin; 0AD. Available from: https://www.onelogin.com/learn/otp-totp-hotp.

[39] M’Raihi D, Bellare M, Hoornaert F, Naccache D, Ranen O. Hotp: An hmac-based one-time password algorithm. The Internet Society, Network Working Group RFC4226. 2005;.

[40] Yin X, He J, Guo Y, Han D, Li KC, Castiglione A. An efficient two-factor authentication scheme based on the Merkle Tree. Sensors. 2020;20(20):5735. doi: 10.3390/s20205735 33050225 PMC7599477

[41] Yoon S, Kim B, Kang Y, Choi D. PUF-based Authentication Scheme for IOT devices. 2020 International Conference on Information and Communication Technology Convergence (ICTC). 2020.

[42] Braeken A. PUF based authentication protocol for IoT. Symmetry. 2018;10(8):352. doi: 10.3390/sym10080352

[43] Wallrabenstein JR. Practical and secure IOT device authentication using physical unclonable functions. 2016 IEEE 4th International Conference on Future Internet of Things and Cloud (FiCloud). 2016.

[44] Akgün M, Çaǧlayan MU. Towards scalable identification in RFID systems. Wireless Personal Communications. 2016;86(2):403–421. doi: 10.1007/s11277-015-2936-7

[45] Bicakci K, Baykal N. Infinite length hash chains and their applications. Proceedings Eleventh IEEE International Workshops on Enabling Technologies: Infrastructure for Collaborative Enterprises. 2002. doi: 10.1109/enabl.2002.1029989

[46] Babkin S, Epishkina A. Authentication protocols based on one-time passwords. 2019 IEEE Conference of Russian Young Researchers in Electrical and Electronic Engineering (EIConRus). 2019.

[47] Akgün M, Çaǧlayan MU. Providing destructive privacy and scalability in RFID systems using PUFs. Ad Hoc Networks. 2015;32:32–42.

[48] Verbauwhede I, Maes R. Physically unclonable functions: manufacturing variability as an unclonable device identifier. In: Proceedings of the 21st edition of the great lakes symposium on Great lakes symposium on VLSI; 2011. p. 455–460.

[49] Wen Y, Lao Y. Efficient fuzzy extractor implementations for PUF based authentication. In: 2017 12th International Conference on Malicious and Unwanted Software (MALWARE); 2017. p. 119–125.

[50] Aung PP, Mashiko K, Ismail NB, Yee OC. Evaluation of SRAM PUF characteristics and generation of stable bits for IOT Security. Advances in Intelligent Systems and Computing. 2019; p. 441–450.

[51] Taniguchi M, Shiozaki M, Kubo H, Fujino T. A stable key generation from PUF responses with a Fuzzy Extractor for cryptographic authentications. In: 2013 IEEE 2nd Global Conference on Consumer Electronics (GCCE); 2013. p. 525–527.

[52] Li Y, Shen J, Liu W, Zou W. A survey on side-channel attacks of strong PUF. In: International Conference on Artificial Intelligence and Security. Springer; 2020. p. 74–85.

[53] Cao Y, Zhao X, Ye W, Han Q, Pan X. A compact and low power RO PUF with high resilience to the EM side-channel attack and the SVM modelling attack of wireless sensor networks. Sensors. 2018;18(2):322. doi: 10.3390/s18020322 29360790 PMC5856110

[54] Rührmair U, Sehnke F, Sölter J, Dror G, Devadas S, Schmidhuber J. Modeling attacks on physical unclonable functions. In: Proceedings of the 17th ACM conference on Computer and communications security; 2010. p. 237–249.

[55] Santikellur P, Bhattacharyay A, Chakraborty RS. Deep learning based model building attacks on arbiter PUF compositions. Cryptology ePrint Archive. 2019;.

[56] Ruhrmair U, Solter J. PUF modeling attacks: An introduction and overview. In: 2014 Design, Automation & Test in Europe Conference & Exhibition (DATE); 2014.

[57] Vijayakumar A, Patil VC, Prado CB, Kundu S. Machine learning resistant strong PUF: Possible or a pipe dream? In: 2016 IEEE international symposium on hardware oriented security and trust (HOST). IEEE; 2016. p. 19–24.

[58] Xu C, Zhang J, Law MK, Jiang Y, Zhao X, Mak PI, et al. Modeling Attack Resistant Strong PUF Exploiting Obfuscated Interconnections With <0.83 In: 2021 IEEE Asian Solid-State Circuits Conference (A-SSCC); 2021. p. 1–3.

[59] Lim D, Lee JW, Gassend B, Suh GE, Van Dijk M, Devadas S. Extracting secret keys from integrated circuits. IEEE Transactions on Very Large Scale Integration (VLSI) Systems. 2005;13(10):1200–1205. doi: 10.1109/TVLSI.2005.859470

[60] Zeitouni S, Vliegen J, Frassetto T, Koch D, Sadeghi AR, Mentens N. Trusted configuration in cloud FPGAs. In: 2021 IEEE 29th Annual International Symposium on Field-Programmable Custom Computing Machines (FCCM). IEEE; 2021. p. 233–241.

[61] Zhang J, Qu G. Physical unclonable function-based key sharing via machine learning for IoT security. IEEE Transactions on Industrial Electronics. 2019;67(8):7025–7033. doi: 10.1109/TIE.2019.2938462

[62] Robinson A, Steinwandt R. Group key establishment with physical unclonable functions. Journal of Information and Optimization Sciences. 2019;40(1):69–80. doi: 10.1080/02522667.2017.1417728

[63] Guajardo J, Kumar SS, Schrijen GJ, Tuyls P. FPGA intrinsic PUFs and their use for IP protection. In: International workshop on cryptographic hardware and embedded systems. Springer; 2007. p. 63–80.

[64] Holcomb DE, Burleson WP, Fu K. Power-up SRAM state as an identifying fingerprint and source of true random numbers. IEEE Transactions on Computers. 2008;58(9):1198–1210. doi: 10.1109/TC.2008.212

[65] Maurya PK, Bagchi S. A secure PUF-based unilateral authentication scheme for RFID system. Wireless Personal Communications. 2018;103(2):1699–1712. doi: 10.1007/s11277-018-5875-2

[66] Boyapally H, Mathew P, Patranabis S, Chatterjee U, Agarwal U, Maheshwari M, et al. Safe is the new smart: PUF-based authentication for load modification-resistant smart meters. IEEE Transactions on Dependable and Secure Computing. 2020;.

[67] Mahalat MH, Karmakar D, Mondal A, Sen B. PUF based Secure and Lightweight Authentication and Key-Sharing Scheme for Wireless Sensor Network. ACM Journal on Emerging Technologies in Computing Systems (JETC). 2021;18(1):1–23. doi: 10.1145/3466682

[68] Aubel PV, Bernstein DJ, Niederhagen R. Investigating SRAM pufs in large cpus and gpus. In: International Conference on Security, Privacy, and Applied Cryptography Engineering. Springer; 2015. p. 228–247.

[69] Gotze KC, Li J, Iovino GM. Fuse attestation to secure the provisioning of secret keys during integrated circuit manufacturing; 2014.

[70] Gotze KC, Iovino GM, Li J. Secure provisioning of secret keys during integrated circuit manufacturing; 2017.

[71] Horovitz K, Kenny R. Intel FPGA secure device manager. Intel Corporation, Programmable Solutions Group (formerly Altera) San Jose …; 2018.

[72] Menhorn N. External secure storage using the PUF. Xilinx, San Jose, CA, USA, Application Note XAPP1333 (v1 0) June 26. 2018;.

[73] Sadeghi AR, Visconti I, Wachsmann C. PUF-enhanced RFID security and privacy. In: Workshop on secure component and system identification (SECSI). vol. 110; 2010.

[74] Mills DL. Internet time synchronization: the network time protocol. IEEE Transactions on Communications. 1991;39(10):1482–1493. doi: 10.1109/26.103043

[75] Dierks T, Rescorla E. The Transport Layer Security (TLS) Protocol Version 1.2; 2008. RFC 5246 (Proposed Standard). Available from: http://www.ietf.org/rfc/rfc5246.txt.

[76] Halderman JA, Schoen SD, Heninger N, Clarkson W, Paul W, Calandrino JA, et al. Lest we remember: cold-boot attacks on encryption keys. Commun ACM. 2009;52(5):91–98. doi: 10.1145/1506409.1506429

[77] Azees M, Vijayakumar P, Jegatha Deborah L. Comprehensive survey on security services in vehicular ad-hoc networks. IET Intelligent Transport Systems. 2016;10(6):379–388. doi: 10.1049/iet-its.2015.0072

[78] Rajasekaran AS, Islam Satti M, et al. An Anonymous Signature-Based Authentication and Key Agreement Scheme for Vehicular Ad Hoc Networks. Security and Communication Networks. 2022;2022.

[79] Azees M, Vijayakumar P, Karuppiah M, Nayyar A. An efficient anonymous authentication and confidentiality preservation schemes for secure communications in wireless body area networks. Wireless Networks. 2021;27(3):2119–2130. doi: 10.1007/s11276-021-02560-y

